# Preventing maternal phenylketonuria (PKU) syndrome: important factors to achieve good metabolic control throughout pregnancy

**DOI:** 10.1186/s13023-021-02108-5

**Published:** 2021-11-18

**Authors:** Carmen Rohde, Alena Gerlinde Thiele, Christoph Baerwald, Rudolf Georg Ascherl, Dinah Lier, Ulrike Och, Christina Heller, Alexandra Jung, Kathrin Schönherr, Monika Joerg-Streller, Simone Luttat, Sabine Matzgen, Tina Winkler, Stefanie Rosenbaum-Fabian, Oxana Joos, Skadi Beblo

**Affiliations:** 1 Hospital for Children and Adolescents, Centre for Pediatric Research Leipzig (CPL), Department of Women and Child Health, University Hospital, University of Leipzig, Leipzig, Germany; 2 University Hospital Internal Medicine, University of Leipzig, Leipzig, Germany; 3 Department of Pediatrics, Klinikum Am Steinenberg, Reutlingen, Germany; 4 Pediatrics Department of the University Clinic, Münster, Germany; 5 Department for Inborn Metabolic Diseases, Children’s and Adolescents’ Hospital, University of Erlangen-Nürnberg, Erlangen, Germany; 6 Center of Excellence for Rare Metabolic Diseases, Charité, University Medicin, Berlin, Germany; 7 Centre for Inborn Metabolic Disorders, Department of Neuropediatrics, Jena University Hospital, Jena, Germany; 8 Clinic for Pediatrics, Inherited Metabolic Disorders, Medical University Innsbruck, Innsbruck, Austria; 9 Clinic for Pediatrics, University Magdeburg, Magdeburg, Germany; 10 Department for General Pediatrics, Metabolic Unit, University Clinic of the the Justus Liebig University, Giessen, Germany; 11 Klinikum Cottbus, Cottbus, Germany; 12 Center of Pediatrics and Adolescent Medicine, University Hospital, Freiburg, Germany; 13 University Hospital, Greifswald, Germany

**Keywords:** Maternal PKU syndrome, Phenylketonuria, Training program, mPKU, Maternal PKU, Pregnancy, PKU

## Abstract

**Background:**

Insufficient metabolic control during pregnancy of mothers with phenylketonuria (PKU) leads to maternal PKU syndrome, a severe embryo-/fetopathy. Since maintaining or reintroducing the strict phenylalanine (Phe) limited diet in adults with PKU is challenging, we evaluated the most important dietary and psychosocial factors to gain and sustain good metabolic control in phenylketonuric women throughout pregnancy by a questionnaire survey with 38 questions concerning therapy feasibility. Among them, the key questions covered 5 essential items of PKU care as follows: General information about maternal PKU, PKU training, diet implementation, individual metabolic care, personal support. In addition, all participating PKU mothers were asked to estimate the quality of their personal metabolic control of the concluded pregnancies. 54 PKU mothers with 81 pregnancies were approached at 12 metabolic centers in Germany and Austria were included. According to metabolic control, pregnancies of PKU women were divided in two groups: group “ideal” (not more than 5% of all blood Phe concentrations during pregnancy > 360 µmol/l; n = 23) and group “suboptimal” (all others; n = 51).

**Results:**

The demand for support was equally distributed among groups, concerning both amount and content. Personal support by the direct social environment (partner, family and friends) (“suboptimal” 71% vs “ideal” 78%) as well as individual metabolic care by the specialized metabolic center (both groups around 60%) were rated as most important factors. The groups differed significantly with respect to the estimation of the quality of their metabolic situation (p < 0.001). Group “ideal” presented a 100% realistic self-assessment. In contrast, group “suboptimal” overestimated their metabolic control in 53% of the pregnancies. Offspring of group “suboptimal” showed clinical signs of maternal PKU-syndrome in 27%.

**Conclusion:**

The development of training programs by specialized metabolic centers for females with PKU in child bearing age is crucial, especially since those mothers at risk of giving birth to a child with maternal PKU syndrome are not aware of their suboptimal metabolic control. Such programs should provide specific awareness training for the own metabolic situation and should include partners and families.

**Supplementary Information:**

The online version contains supplementary material available at 10.1186/s13023-021-02108-5.

## Background

Phenylketonuria (PKU) is the most frequent inborn error of amino acid metabolism. Untreated, it leads to severe psychomotor retardation. Since establishment of newborn screening in the 1970s and early and continuous treatment throughout life patients develop normally [1–4]. Most women with PKU live a normal life and family planning becomes an important issue. In this context, ideal metabolic control during pregnancy is absolutely necessary to prevent maternal PKU syndrome, a severe embryo-/fetopathy. Characteristic signs are microcephaly, mental retardation, growth retardation and birth defects including congenital heart disease [3, 5, 6]. Phenylalanine (Phe) is an essential amino acid and is shuttled actively from the mother to the unborn by the placenta [1, 7]. Therefore, fetal blood Phe concentrations are up to twice as high as their mothers’ [1, 7]. High maternal Phe concentrations during conception and pregnancy severely impair organogenesis and growth, and maternal PKU syndrome ensues if the mother’s phenylalanine (Phe) concentrations rise above 360 µmol/l during pregnancy. To prevent the birth of a child with maternal PKU syndrome, mothers must therefore keep their plasma Phe concentration between 120 and 360 µmol/l [8–10]. Unplanned pregnancies should be strictly avoided.

It is a challenge to achieve good metabolic control throughout pregnancy. After mandatory strict dietary treatment during childhood with ideal blood Phe concentrations between 42 and 240 (− 360 µmol/l), higher levels are accepted from puberty onwards (up to 600 or 900 µmol/l) [2, 3]. It is recommended that all women with PKU considering pregnancy resume a strict diet.

We hypothesized that currently implemented structures are insufficient to fulfill patients’ needs in this sensitive situation. Specialized PKU care for adults, especially with respect to care during pregnancy, is still scarce [4, 11, 12] and most gynecologists and obstetricians are unfamiliar with this disease and the effects of typical pregnancy related issues on the metabolic control in PKU. Attempts to improve care include home visitation programs [13], hospitalization during pregnancy [14], or accompanying psychotherapy [15], all with inconclusive results.

To establish a structured program for PKU pregnancy care, it is important to identify factors which are helpful for women with PKU to restart a strict low-phe diet before conception and during pregnancy. We therefore performed a cross-sectional multicenter evaluation at 12 German speaking metabolic centers with the focus on women’s needs to support this important phase of life.

## Methods

The metabolic center of Leipzig, Germany, designed a retrospective, multi-center evaluation. It was approved by the ethics committee at the University of Leipzig Medical Faculty and local ethics committees of participating centers. The study was registered with the German Clinical Trial register (registration numbers 027-16-01022016, DRKS 00013706) at the International Clinical Trials Registry Platform. At the participating centers (Berlin, Cottbus, Erlangen-Nürnberg, Freiburg, Giessen, Greifswald, Jena, Leipzig, Magdeburg, Münster, Reutlingen, all in Germany, and Innsbruck, Austria), all mothers with PKU were approached at routine clinic visits between April 2016 and January 2018. Participants gave written informed consent. All pregnancies were already concluded at the time of answering a two-part multiple choice questionnaire, specifically designed for this purpose (Additional file 1). One case of termination of pregnancy due to antenatal evidence of major malformation has been reported from one of the treatment centers; for lack of any further information, this case could not be included. Mothers with more than one pregnancy were asked to answer the questionnaire separately for each pregnancy.

Mothers with PKU were asked to answer 36 general questions about former and current treatment and diet implementation as well as pregnancy outcome. In addition, two extended key questions concerning diet feasibility during pregnancy (“*What was helpful for you keeping the diet during pregnancy?*” and “*What have you missed during pregnancy?*”*)* were provided. Here, 22 answering options were offered, and patients could mark as many options as they felt appropriate (Additional file 1). Answering options marked as “helpful” or “missed” were summed up to “important factors” and reasserted to cover 5 essential items as follows: General information about matsternal PKU, PKU training, diet implementation, individual metabolic care, personal support. As multiple answers were possible, the given numbers are not adding up to the total n, and the given percentages are not adding up to 100. Additionally, participants could provide personal statements, almost only used by single patients repeating already offered answers within the multiple choice part.

To gain additional information about knowledge and awareness of metabolic control during pregnancy, the question “*How would you estimate your metabolic control during pregnancy?*” was included in the questionnaire. Four responses were offered: “*almost all (*> *80%) measured Phe concentrations were* < *240 µmol/l*”, “*more than half of all measured Phe concentrations were* < *240 µmol/l*”*,* “*less than half of all measured Phe concentrations were* < *240 µmol/l*”*,* and “*almost all (*> *80%) measured Phe concentrations were* > *240 µmol/l*”. We chose the cutoff of 240 µmol/l because this target value was widely recommended at the time of the mothers’ pregnancies [1, 7]. Of note, all pregnancies had already been concluded. Therefore, these data represent the mothers own retrospective impression of metabolic control.

The second part of the questionnaire included data about metabolic control before and during pregnancy. These data were provided by the treating metabolic centers.

According to metabolic control, we divided pregnancies in two subgroups: group “ideal” presented metabolic control with no more than 5% of all blood Phe concentrations during pregnancy above 360 µmol/l; group “suboptimal” included all other pregnancies.

Statistical analysis was performed using SPSS 25 (IBM). Group comparisons were performed with respect to patient characteristics, metabolic control, as well as self-estimation of own metabolic control during pregnancy and “important factors” for dietary control. Since data were not normally distributed, Mann–Whitney-U test was used to perform group comparisons, with weighting according to group sizes. Significance was assumed for p < 0.05.

## Results

65 mothers with PKU could be approached by the respective treatment centers, 54 were willing to answer the questionnaire for a total number of 81 pregnancies. 7 questionnaires were excluded from analysis: 2 because patients received cofactor treatment with sapropterin dihydrochloride during pregnancy and another 5 where no laboratory data were available (technical loss of data in the respective treatment center). Altogether, 74 evaluable questionnaires could be analyzed for either the first (n = 50), second (n = 21) or third (n = 3) pregnancy.

23 pregnancies (31%) could be assigned to the group “ideal”. With one exception, all of these pregnancies were planned. The remaining 51 pregnancies (69%) were assigned to the group “suboptimal”, of which 11 pregnancies were unplanned. In case of repeated pregnancies, each of these was assorted to the respective category. All multiple pregnancies were found to be in the same group with four exceptions. Three mothers showed better metabolic control during the first compared to subsequent pregnancies.

Patient characteristics and data about metabolic control as well as offspring outcome of both groups are shown in Tables 1 and 2. The groups differed significantly regarding mean blood Phe values (p < 0.001) and mean percentage (%) of Phe values above the therapeutic range (p < 0.001) (Table 2). Mothers of group “ideal” had a significantly higher level of education (Table 1). No differences could be found regarding the following parameters: Phe tolerance, frequency of blood Phe testing, kind of diet implementation (calculating or estimating Phe consumption), and perceived burden of keeping the diet, number of pregnancies, and mothers’ mean age at the beginning of pregnancy (Table 1). However, offspring of mothers of group “suboptimal” showed suspicious signs for maternal PKU syndrome in 27%, whereas none of the offspring from mothers with ideal metabolic control seems to be affected.

**Table 1:** Patient characteristics

	Group "ideal"	Group "suboptimal"	p (Mann–Whitney-U)
	n = 23	n = 51	
*Patients: n (%)*			
Planned pregnancies	22 (96)	40 (78)	1
Unplanned pregnancies	1 (4)	11 (22)	1
*Pregnancies: n (%)*			
First	18 (78)	31 (61)	1
Second	5 (22)	16 (31)	1
Third	0 (0)	4 (8)	
*Phe tolerance before pregnancy (mg/day)*			
Mean (SD)	587 (235)	572 (264)	0.798
Median (IQR)	560 (400–787)	500 (362–800)	
Range	250–950	300–1200	
*Phe-tolerance known before pregnancy: n (%)*			
Known	12 (52)	28 (55)	1
Unknown	11 (48)	23 (45)	1
*Blood Phe-analyses during pregnancy (n)*			
Mean (SD)	38 (14)	33 (12)	
Median (IQR)	35 (29–42)	34 (28–40)	0.345
Range	17–79	5–66	
*Diet implementation: n (%)*			
Calculation of all foods	2 (9)	10 (20)	0.320
Calculation of all foods except fruit, vegetables and low protein foods	0 (0)	1 (2)	1
No consumption of protein-rich foods and estimation of Phe-intake	7 (30)	14 (27)	1
Consumption of protein-rich foods and estimation of Phe-intake	9 (39)	12 (24)	0.265
None	5 (22)	14 (27)	0.776
*Feeling about diet: n (%)*			
Very hard	2 (9)	5 (10)	1
Hard	4 (17)	13 (25)	0.558
Easy	14 (57)	22 (43)	0.211
Very easy	4 (17)	10 (20)	1
*Age of mothers at beginning of pregnancy (years)*			
Mean (SD)	29.5 (4,3)	28.90	
Median (IQR)	29 (26–33)	29 (26–32)	0.258
Range	21–38	19–39	
*Mother*’*s highest achieved degree of school education: n (%)*			
Primary school	0 (0)	0 (0)	1
School for mentally handicapped children	0 (0)	2 (4)	0.566
9th grade	0 (0)	6 (12)	0.168
10th grade	10 (43)	29 (57)	0.323
12th grade	13 (57)	14 (27)	0.013*

**Table 2:** Metabolic control

	Group "ideal"	Group "suboptimal"	p
	n = 23	n = 51	(Mann–Whitney U)
*Blood Phe concentration during pregnancy (µmol/l)*			
Range of all measurements	6–762	8–1195	
Mean of mean values (SD)	158 (35)	292 (160)	
Median of mean values (IQR)	154 (123–188)	245 (202–292)	< 0.001
Range of mean values	101–225	115–882	
*Phe* > *360 µmol/l: n (%)*			
Mean (SD)	1.3 (2)	26 (25)	
Median (IQR)	0 (0–3)	34 (28–40)	< 0.001
range	0–5	6–100	
Clinical signs of maternal PKU syndrom: n* (%)	0	14 (27)	n.a
Heart defect	0	1 (2)	
Microcephaly	0	4 (8)	
Microsomia	0	2 (4)	
Behavior abnormalitie	0	7 (14)	
Developmental delay	0	6 (12)	
Special school	0	6 (12)	

^*^7 children suffering from more than one clinical sign suspicious of maternal PKU syndrome

**Fig. 1:**
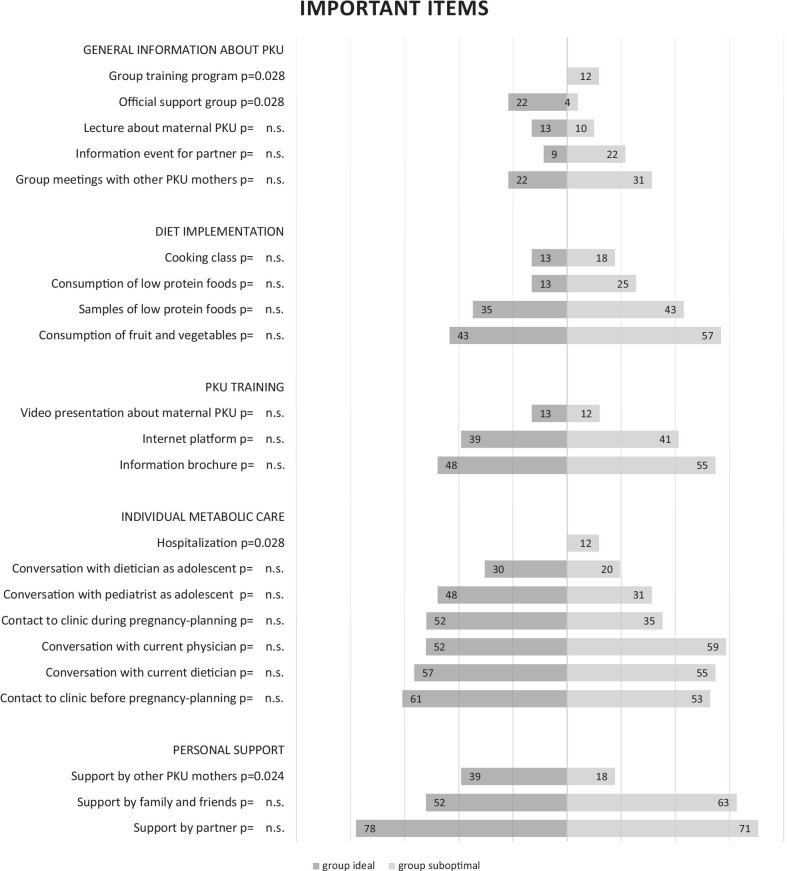
Items designated as “important” (missing / helpful factors) by group “ideal” or “suboptimal”. Bars represent the percentages of patients from group “ideal” respectively “suboptimal” choosing items as important. P for Mann–Whitney-U test, n. s. = not significant

Important items to maintain metabolic control throughout pregnancy and their individual ranking are shown in Fig. 1 for subgroups “ideal” and “suboptimal”.

Both groups seem to need an equal amount of support. Most frequently mentioned important factor in all proposed items in both groups was identified as “*support by my partner*”*,* next to “*individual metabolic care*” by the metabolic center.

Comparing the frequency of answering options and, therefore, the importance of different factors between groups, no significant differences could be found with four exceptions. Patient group “suboptimal” mentioned significantly more often “*group training*” and “*hospitalization*” as important factors.

In contrast, group “ideal” mentioned significantly more often an “*official support group*” and “*support by other PKU mothers*”.

The data about their retrospective self-estimation of metabolic control during pregnancy at the time of the questionnaire is shown in Table 3. While the subgroup “ideal” showed a realistic estimation, the subgroup “suboptimal” overestimated their own metabolic control and guessed it being far better than the data proved it to be in 53% of the pregnancies. This difference between groups regarding self-assessment was significant (p < 0.001) (Table 3).

**Table 3:** Estimation of own metabolic control during pregnancy

	Group "ideal"		Group "suboptimal"		P (Mann–Whitney-U test)
	n = 23		n = 51		
	Realistic n (%)	Overestimation n (%)	Realistic n (%)	Overestimation n (%)	
Almost all (> 80%) blood Phe concentrations < 240 µmol/l	16 (70)	0	9 (18)	18 (35)	
More than half (> 50%) of all blood Phe concentrations < 240 µmol/l	5 (18)	1 (4)	8 (16)	9 (18)	
Less than half (< 50%) of all blood Phe concentrations < 240 µmol/l	0	0	5 (10)	0	
Almost all (> 80%) blood Phe concentrations > 240 µmol/l	0	0	0	0	
Total	21 (88)	1 (4)	22 (44)	27 (53)	< 0.001
Question not answered	2 (8)	2 (4)			

Concerning the nutritional aspects in the questionnaire, no differences in importance of specific special low protein foods for maintaining metabolic control could be revealed between groups. Therefore, only cumulative data is shown in Tables 4 and 5. In 96% of the patients special low protein cereal foods (bread and buns) were the most helpful products to create a sensible dietary plan.

**Table 4:** Most helpful low protein food

	n = 74 (%)
Bread, buns	71 (96)
Milk replacer	43 (58)
Cakes, cookies	19 (26)
Meet replacer	15 (20)
Cheese replacer	13 (18)
Sweets, chocolate, bars	12 (16)
Convenient foods	9 (12)
Noodles, pasta	8 (11)

**Table 5:** Preferred pharmaceutical form of amino acid mixture

	n = 74 (%)
Non-flavoured powder, box	23 (31)
Ready-to-drink mixes	21 (28)
Pills	20 (27)
Non-flavoured powder, sachets	16 (22)
Flavoured powder, sachets	7 (9)
Flavoured powder, box	2 (3)
Ready-to-eat bars	2 (3)
"Pudding"	1 (1)

This was followed by low protein milk replacement, whereas all other foods are obviously of far less importance. Only 11% (n = 8) of the PKU women mentioned low protein pasta (Table 4).

The preferred choice of Phe free amino acid mixtures is shown in Table 5. Again, no differences between the subgroups “ideal” and “suboptimal” were found. The data showed that the preferred amino acid mixtures were non-flavored powders and ready-to-drink mixes. Except for amino-acid pills, special designed products such as bars or “puddings” were unpopular (Table 5).

## Discussion

Gaining and maintaining good metabolic control while planning and during pregnancy is the most important factor for women with PKU who wish to give birth to healthy children. Constant blood Phe concentrations < 360 µmol/l are the best prevention of maternal PKU syndrome [4, 6, 8, 9, 16]. However, adolescents and adults with PKU are often not compliant to the prescribed diet, irrespectively of being male or female [17–20]. Programs to help women with PKU to achieve sufficient metabolic control during pregnancy have been demanded for decades [8, 21, 22]. Attempts include home visitation programs [13] hospitalization during pregnancy [14], or accompanying psychotherapy [15], all with inconclusive results. The study presented here aimed to identify most important factors to gain and sustain good metabolic control in phenylketonuric women throughout pregnancy, assuming that the current programs of health care systems might be off target or insufficient. Using an especially designed two-part multiple choice questionnaire, we could identify factors, which mothers indicated as important to support good metabolic control during 74 concluded pregnancies.

In a previous study, we found that a cohort of well treated PKU patients show about 30% of blood Phe concentrations above the recommended range [23], which is in accordance with data from another recent investigation [26]. Clearly, this amount of Phe-concentrations off target seemed to us to be insufficient during pregnancy. In contrast, to expect that not a single sample would display elevated blood Phe concentrations is not a realistic approach. As a compromise, we allowed up to 5% of elevated blood Phe concentrations during pregnancy and still defined this as “ideal” metabolic control. Accordingly, we divided the pregnancies into two groups “ideal” and “suboptimal”.

Phe tolerance, frequency of blood Phe testing, dietary management, burden of keeping the diet, number of pregnancies and mothers’ mean age at the beginning of pregnancy were comparable between these groups.

However, self-estimation of metabolic control shows significant deficiencies in the group “suboptimal”, and signs of maternal PKU syndrome could be revealed in almost 30% of their offspring, whereas none of the children born to mothers with “ideal” metabolic control was affected. Interestingly, group “ideal” showed significantly higher school education compared to group “suboptimal”.

Concerning most important factors to support good metabolic control, both groups specified “*personal support*”*,* especially “*support by my partner*” and “*support by my family and friends*”. This was followed by specialty care offered by the metabolic center (“*contact to a metabolic center before planning a pregnancy*”*,* “*informative conversation with current dietician*”*,* “*informative conversation with current physician*”).

Other options, such as specific printed information material or samples of special low-protein food, were less important. Only the group “suboptimal” listed “*group training*” and “*hospitalization*” as helpful factors. These patients seem to recognize their need to improve metabolic control and therefore are even willing to undergo hospitalization for a while. In contrast group “ideal” was looking for “*official support group*” and contact to “*other PKU mothers*”.

Our most important finding is that a substantial proportion of pregnant PKU patients had an inaccurate self-estimation of their metabolic situation. This accounts for a volatile situation since this overestimation is in coincidence with suboptimal control and signs of maternal PKU syndrome in the offspring. The currently implemented patient education, even in specialized outpatient clinics, seems therefore insufficient.

Establishment of separate clinics for adults geared toward adolescents with PKU can support the process of taking over responsibility for their own disease awareness and treatment [20, 24]. However, the process to become autonomous cannot be performed as a one-way procedure by working only with the patients. Special training of the respective parents on how to allow more responsibility through their children should be implemented – comparable to how it has already been shown in the field of other chronic diseases such as type I diabetes [25]. Differences in general educational levels in patients and their families have to be incorporated in this respect, calling for a maximum of individual support of PKU patients from early childhood onwards not only for their own life but also in preparation for the next generation.

Establishment of clinic visits for adolescents without their parents is an important part towards this development. By this, the transition to the adult medical care can be prepared and started to ensure continuous contact to the metabolic center [26, 27].

Metabolic centers should actively encourage the partner’s participation in clinic visits, educational activities or consultations with dietitians, as personal support by the partner or/and family members was rated as the most important factor in both groups to achieve good metabolic control. Specialized programs approaching partners or couples should be implemented which help to prepare for pregnancy.

As a consequence of this study’s observations, the Leipzig metabolic center has introduced a targeted weekend seminar incorporating most of the aspects discussed here for young PKU women and their partners which already achieves cross-regional attention [28].

The main limitation of the study comprises the restricted variability within the investigated group. Most of the participating PKU women are still under constant specialized care in one of the 12 metabolic centers. PKU women without specialized care or after dropping out of treatment and care could not be reached by the chosen approach. This is of importance, as the risk of giving birth to a child with maternal PKU syndrome is continuously rising, because the number of healthy PKU women reaching childbearing age is increasing [29, 30]. The number of unreported pregnancies is probably high and maternal PKU syndrome in an offspring is not easy to detect as severity varies. Pediatricians caring for children with developmental delay might even not be aware of the mother’s PKU.

## Conclusion

Facing the constantly rising number of children delivered by PKU mothers, the development of training programs for PKU females in child bearing age is crucial. Since the importance of personal communication with partners and families as well as PKU professionals within the specialized healthcare system could not be overruled by other important items, these programs should be tailored according to the specific needs of this patient group, including an early start of transition from pediatric to continuous adult care and education of partners of all young women with PKU.

## Supplementary Information

**Additional file 1**. Survey Maternal Phenylketonuria.

## Data Availability

The datasets used and/or analysed during the current study are available from the corresponding author on reasonable request.

## Abbreviations

PKU: Phenylketonuria
Phe: Phenylalanine
DRKS: Deutsches Register klinischer Studien (German Clinical Trials Register
