# Supplementary material for: Preventing maternal phenylketonuria (PKU) syndrome: important factors to achieve good metabolic control throughout pregnancy
Source: Orphanet J Rare Dis. 2021 Nov 18;16:477. doi: 10.1186/s13023-021-02108-5 (PMC8600879; doi:10.1186/s13023-021-02108-5)
Supplement: Supplementary file 1 — Additional file 1. Survey Maternal Phenylketonuria. [file 13023_2021_2108_MOESM1_ESM.docx]

Klinik und Poliklinik für Kinder- und Jugendmedizin

Ernährungstherapie, Dr. troph. Carmen Rohde

Liebigstraße 20a, 04103 Leipzig

[carmen.rohde@uniklinik-leipzig.de](mailto:carmen.rohde@uniklinik-leipzig.de), Tel. 0341/9726350

Date:______________

Survey Maternal Phenylketonuria

| **To be filled in by metabolic center:**  Please fill in a separate survey for each pregnancy!  **Patient-No: __________________** □ 1. child □ 2. child □ 3. child □ 4. child  **Metabolic center: ____________________** |
| --- |

**PART 1 (to be filled in by the patient)**

**How old is your child presently?**

□ ______ years

**How did you manage your diet before your pregnancy?**

**Please pick the answer which fits most.**

□ Phe-content of all foods was calculated.

□ Phe-content of all foods was calculated, except of special low protein products, fruit and vegetables.

□ Phe-content of all foods was estimated. Protein rich foods as meat, sausages, fish, eggs and diary were not consumed.

□ Phe-content of all foods was not calculated and not estimated. Protein rich foods as meat, sausages, fish, eggs and diary were consumed in very little portions only or not at all.

□ I did not adhere to any specialized diet.

**Did you use BH4 before your pregnancy?**

□ yes

□ no

**Did you use BH4 during your pregnancy?**

□ yes

□ no

**What was your Phe-tolerance or your protein-tolerance before pregnancy?**

□ I do not know

□ about ________ mg Phe/day OR about _______ g protein/day

**How old were you at the begin of pregnancy?**

□ _____ years

**How tall and how heavy were you at the begin of pregnancy?**

□ _____ cm

□ _____ kg

**During which week of pregnancy was your child delivered?**

□ ____ . week of pregnancy

**Were there any complications during or after delivery?**

□ _____ no

□ _____ yes, complication: _______________________________

**How tall and how heavy was your child at birth? What was the circumference of the head?**

□ _____ cm length

□ _____ kg weight

□ _____ cm circumference of the head

**Does your child suffer from any abnormalities?**

□ heart defect

□ small circumference of the head

□ microsomia

□ behavior abnormalities

□ developmental delay

□ early intervention/school for handicapped children

□ none

**Was your general practitioner familiar with PKU?**

□ yes

□ no

**Was your gynecologist familiar with PKU?**

□ yes

□ no

**Was your pregnancy planned or unplanned?**

□ planned

□ unplanned

**How often did you visit the metabolic center before pregnancy?**

□ ____ times

**How often did you visit the metabolic center during pregnancy?**

□ ____ times

**How often did you visit the metabolic center after delivery?**

□ ____ times

**Did you experience nausea often during pregnancy?**

□ yes

□ no

**If yes, when did the nausea stop?**

□ after the first 3 months?

□ after the first 6 months?

**Were you in need of energy supplements(e.g. Duocal) to counteract dietary fatigue and loss of weight?**

□ yes

□ no

**Were any fetal abnormalities observed during pregnancy?**

□ ultrasound

□ growth development

□ other_______________________

**Were you admitted to the hospital during pregnancy to adjust metabolic control?**

□ yes

□ no

**As an adolescent, were you informed about the need to have your Phe-concentration measured and keep it below a certain level?**

□ yes

□ no

**What was helpful in staying on a diet during pregnancy?**

**You can pick as many answers as you want.**

□ Conversation with pediatrist

□ Conversation with current physician

□ Conversation with pediatric dietician

□ Conversation with current dietician

□ Contact to clinic before pregnancy-planning

□ Contact to clinic during pregnancy-planning

□ Group training program

□ Cooking class

□ Hospitalization

□ Lecture about maternal PKU

□ Video about maternal PKU

□ Information brochure

□ Internet platform

□ Samples of low protein foods

□ Consumption of low protein foods

□ Consumption of fruit and vegetables

□ Support of partner

□ Information event for partner

□ Support of family and friends

□ Support of other PKU mothers

□ Group meetings with PKU mothers

□ Official support group

□ ______________________________________________

**What have you missed during pregnancy?**

□ Clarification on maternal PKU by a pediatrician during pregnancy

□ Clarification by a current physician

□ Clarification on maternal PKU by pediatric dietician as an adolescent

□ Clarification by a current dietician

□ Referral to specialized clinic before pregnancy-planning

□ Referral to specialized clinic during pregnancy-planning

□ Group training program

□ Cooking class

□ Hospitalization

□ Lecture about maternal PKU

□ Video about maternal PKU

□ Information brochure

□ Internet platform

□ Samples of low protein foods

□ Consumption of low protein foods

□ Consumption of fruit and vegetables

□ Support of partner

□ Information event for partner

□ Support of family and friends

□ Support of other PKU mothers

□ Group meetings with PKU mothers

□ Official support group

□ _________________________________________________________

**Which special low protein products were helpful to you? You can tick as many answers as you want.**

□ bread, buns

□ cake, cookies

□ sweets, chocolate, bars

□ meat replacement

□ cheese replacement

□ milk replacement

□ ready to use foods

**Which of those foods was most helpful?**

□ bread, buns

□ cake, cookies

□ sweets, chocolate, bars

□ meet replacement

□ cheese replacement

□ milk replacement

□ convenient food

**Which food did you miss most?**

□ ­­­­__________________________

□ __________________________

□ __________________________

**How did you feel, regarding the diet?**

□ It was very hard on me.

□ It was hard on me.

□ It was easy on me.

□ It was very easy on me.

**How many portions of protein substitute did you take during the day?**

□ 1-2 portions

□ 3 portions

□ 4 or more portions

**How did you feel regarding the use of the protein substitute?**

□ It was very hard on me.

□ It was hard on me.

□ It was easy on me.

□ It was very easy on me.

**What kind of protein substitute would you prefere?**

□ non-flavored powder, box

□ flavored powder, box

□ non-flavored powder, sachets

□ flavored powder, sachets

□ pills

□ ready-to-drink mixes

□ ready-to-eat bars

□ „pudding“

□ anything else ________________

**How often have you send in dried blood cards during pregnancy?**

**Which answer fits for you most?**

□ monthly or less

□ every other week

□ weekly

□ several times during the week

**How would you estimate your metabolic control during pregnancy?**

**Only one answer is possible.**

□ almost all measured Phe-concentrations were < 240 µmol/l

□ the majority of all measured Phe-concentrations were < 240 µmol/l

□ the majority of all measured Phe-concentrations were > 240 µmol/l

□ almost all measured Phe-concentrations were > 240 µmol/l

**How would you estimate your metabolic control during pregnancy?**

**(Careful! Other reference values!)**

**Only one answer is possible.**

□ almost all measured Phe-concentrations were < 600 µmol/l

□ the majority of all measured Phe-concentrations were < 600 µmol/l

□ the majority of all measured Phe-concentrations were > 600 µmol/l

□ almost all measured Phe-concentrations were > 600 µmol/l

**What is your highest achieved degree of school education?**

□ primary school

□ school for mentally handicapped children

□ 9th grade

□ 10th grade

□ 12th grade

| **PART 2: to be filled in by the metabolic center**  **How high have the Phe-and Tyr-concentrations been during pregnancy?**   \| date \| Phe in µmol/l \| Tyr in µmol/l \| \| --- \| --- \| --- \| \|  \|  \|  \| \|  \|  \|  \| \|  \|  \|  \| \|  \|  \|  \| \|  \|  \|  \| \|  \|  \|  \| \|  \|  \|  \| \|  \|  \|  \| \|  \|  \|  \| \|  \|  \|  \| \|  \|  \|  \| \|  \|  \|  \| \|  \|  \|  \| \|  \|  \|  \| \|  \|  \|  \| \|  \|  \|  \| \|  \|  \|  \| \|  \|  \|  \| \|  \|  \|  \| \|  \|  \|  \| \|  \|  \|  \| \|  \|  \|  \| \|  \|  \|  \| \|  \|  \|  \| \|  \|  \|  \| \|  \|  \|  \| \|  \|  \|  \| \|  \|  \|  \| \|  \|  \|  \| \|  \|  \|  \| \|  \|  \|  \| \|  \|  \|  \| \|  \|  \|  \| \|  \|  \|  \| \|  \|  \|  \| \|  \|  \|  \| \|  \|  \|  \| \|  \|  \|  \| \|  \|  \|  \| \|  \|  \|  \| \|  \|  \|  \| \|  \|  \|  \| |
| --- | --- | --- | --- | --- | --- | --- | --- | --- | --- | --- | --- | --- | --- | --- | --- | --- | --- | --- | --- | --- | --- | --- | --- | --- | --- | --- | --- | --- | --- | --- | --- | --- | --- | --- | --- | --- | --- | --- | --- | --- | --- | --- | --- | --- | --- | --- | --- | --- | --- | --- | --- | --- | --- | --- | --- | --- | --- | --- | --- | --- | --- | --- | --- | --- | --- | --- | --- | --- | --- | --- | --- | --- | --- | --- | --- | --- | --- | --- | --- | --- | --- | --- | --- | --- | --- | --- | --- | --- | --- | --- | --- | --- | --- | --- | --- | --- | --- | --- | --- | --- | --- | --- | --- | --- | --- | --- | --- | --- | --- | --- | --- | --- | --- | --- | --- | --- | --- | --- | --- | --- | --- | --- | --- | --- | --- | --- | --- | --- | --- |
